# *β*-Catenin-Dependent Signaling Pathway Contributes to Renal Fibrosis in Hypertensive Rats

**DOI:** 10.1155/2015/726012

**Published:** 2015-04-07

**Authors:** Catherina A. Cuevas, Cheril Tapia-Rojas, Carlos Cespedes, Nibaldo C. Inestrosa, Carlos P. Vio

**Affiliations:** ^1^Department of Physiology, Faculty of Biological Sciences, Pontificia Universidad Catolica de Chile, Alameda 340, 8331150 Santiago, Chile; ^2^Department of Cellular and Molecular Biology, Faculty of Biological Sciences, Pontificia Universidad Catolica de Chile, Alameda 340, 8331150 Santiago, Chile; ^3^Center for Aging and Regeneration CARE-Chile UC, Pontificia Universidad Catolica de Chile, Alameda 340, 8331150 Santiago, Chile

## Abstract

The mechanism of hypertension-induced renal fibrosis is not well understood, although it is established that high levels of angiotensin II contribute to the effect. Since *β*-catenin signal transduction participates in fibrotic processes, we evaluated the contribution of *β*-catenin-dependent signaling pathway in hypertension-induced renal fibrosis. Two-kidney one-clip (2K1C) hypertensive rats were treated with lisinopril (10 mg/kg/day for four weeks) or with pyrvinium pamoate (Wnt signaling inhibitor, single dose of 60 ug/kg, every 3 days for 2 weeks). The treatment with lisinopril reduced the systolic blood pressure from 220 ± 4 in 2K1C rats to 112 ± 5 mmHg (*P* < 0.05), whereas the reduction in blood pressure with pyrvinium pamoate was not significant (212 ± 6 in 2K1C rats to 170 ± 3 mmHg, *P* > 0.05). The levels of collagen types I and III, osteopontin, and fibronectin decreased in the unclipped kidney in both treatments compared with 2K1C rats. The expressions of *β*-catenin, p-Ser9-GSK-3beta, and the *β*-catenin target genes cyclin D1, c-myc, and bcl-2 significantly decreased in unclipped kidney in both treatments (*P* < 0.05). In this study we provided evidence that *β*-catenin-dependent signaling pathway participates in the renal fibrosis induced in 2K1C rats.

## 1. Introduction

Hypertension is a major risk factor for development and progression of chronic kidney disease [1]. The main consequence of the untreated hypertension is the chronic renal injury including vascular, glomerular, and tubulointerstitial injuries. Renal fibrosis is a hallmark of chronic hypertensive disease. Moreover, in animals and patients with chronic hypertension, the decline on renal function is correlated with the degree of renal fibrosis leading to end-organ failure [2]. Different factors have been involved in the pathophysiology of hypertension including the local overactivation of renin-angiotensin system (RAS) mainly by angiotensin II (Ang II) actions [3]. Studies using the model of Ang II-dependent hypertension have showed extensive glomerular and tubulointerstitial fibrosis [4]. In fact, it has been shown that the hypertension induced in 2K1C Goldblatt model is a consequence of increase in tissue Ang II content in both acute and chronic 2K1C animals [5].

Ang II works as a systemic vasoconstrictor, as a modulator of renal microcirculation, and as a regulator of sodium tubular transport [6]. However, Ang II seems to be a main contributor to progressive renal fibrosis through mechanisms participating in the production of chemotactic and profibrotic factors, recruitment of macrophages and myofibroblasts, and extracellular matrix protein production [7]. Chronic infusion of Ang II in murine models induces vascular and renal injuries with interstitial infiltration and increased collagen deposition [8, 9]. Additionally, it has been shown that Ang II is a potent upregulator of osteopontin (OPN), which acts as a chemoattractant molecule to promote fibroblast proliferation [10]. Moreover,* in vitro* studies have shown that Ang II stimulates fibronectin, TGF-*β*, CTGF, and PAI-1 synthesis [11]. Several models of kidney disease in both rodents and humans display local induction of the angiotensin converting enzyme (ACE) which is a well-known enzyme capable of forming Ang II from Ang I, providing an explanation for the elevated renal levels of Ang II in several pathological conditions [12]. In fact, ACE inhibitors (ACEi) or Ang II receptor antagonists are widely used for the treatment of the hypertension as they are known to have antifibrotic effects on the kidney [13].

The Wnt/*β*-catenin signaling pathway participates in organogenesis and tissue homeostasis, and its deregulation has been linked in the pathogenesis of human diseases, including cancers and degenerative diseases [14]. In the cell membrane, Wnt ligands transmit their signal through the interaction with Frizzled receptor and LRP5/6 coreceptor, initiating a series of molecular events leading to an increase in cytosolic *β*-catenin. The nuclear translocation of *β*-catenin allows its association with T-cell factor/lymphoid enhancer factor (TCF/LEF) transcription factors initiating the transcription of *β*-catenin-dependent target genes [15]. In the absence of Wnt ligands, the complex formed by APC-CKI-Axin-GSK-3*β* is assembled to form the destruction complex of *β*-catenin. Once the complex is assembled, the phosphorylation of *β*-catenin by glycogen synthase kinase-3*β* (GSK-3*β*) is the signal for ubiquitination and degradation by the proteasome system [14]. There are recent studies suggesting that the Wnt signaling pathway, mainly the *β*-catenin-dependent signaling, is altered and might have a role in fibrotic kidneys after obstructive injury (unilateral ureteral obstruction) [16]. In addition, it has been showed that several profibrotic genes, such as fibronectin, collagens, and OPN, are *β*-catenin target genes in different cellular contexts [17–19]. However, whether the *β*-catenin signaling participates in the renal interstitial fibrosis induced by Ang II remains to be investigated. Recently, it was reported that *β*-catenin signaling was potently targeted by pyrvinium pamoate [20], an anthelmintic drug. This study was conducted in 2K1C Goldblatt hypertensive rats with the hypothesis that *β*-catenin-dependent signaling pathway contributes to renal fibrosis induced in this model.

## 2. Methods

### 2.1. Animals

All animal studies were performed in accordance with the Guiding Principles in the Care and Use of the Laboratory Animals for the American Physiological Society and were approved by the Ethics Committee of Animal Care of the Pontificia Universidad Católica de Chile. Animals were maintained at constant room temperature with a 12 h light/dark cycle in the institutional animal care facilities (PHS, NIH, OLAW, Animal Welfare Assurance #A5848-01) with free access to food and water.

### 2.2. 2K1C Goldblatt Hypertensive Rats and Treatments

Male Sprague-Dawley rats of 100–125 g were anesthetized with ketamine : xylazine i.p. (25 : 2.5 mg/kg) and a silver clip (0.20 mm) was placed around the left renal artery through a left flank incision. Animals were randomly divided into four groups (4 animals per group) according to the treatment: sham operated control, 2K1C rats without treatment, 2K1C rats treated with lisinopril (10 mg/kg/day) by oral gavage for four weeks starting from fourth week after surgery, and 2K1C rats treated with pyrvinium pamoate (60 *μ*g/kg, single dose every 3 days) by oral gavage for 2 weeks starting from sixth week after surgery. The experiments with pyrvinium pamoate were subsequently performed to lisinopril experiments; therefore, each setting has its own sham control and 2K1C group. Pyrvinium pamoate was purchased from Sigma-Aldrich Co. (St. Louis, MO) and lisinopril was obtained from Recalcine CFR Pharmaceuticals. Systolic blood pressure (SBP) was measured at the end of experiments in conscious rats before the sacrifice using the tail-cuff plethysmography with a Grass polygraph. The rats were sacrificed at the end of 8th week after surgery by overdose of ketamine : xylazine. An independent group of 2K1C and sham rats (*n* = 8 per group) was formed with the purpose of monitoring the level of SBP throughout the experimental period (0–8 weeks). The unclipped kidney was immediately excised and frozen at −80°C or fixed in Bouin's solution and processed for conventional histology, immunofluorescence, and immunohistochemistry.

### 2.3. Western Blot Analysis

Proteins were extracted from whole kidneys sections and were homogenized in RIPA buffer (50 mM Tris-HCl, 150 mM NaCl, 1% NP-40, 0.5% sodium deoxycholate, and 0.1% SDS) supplemented with protease and phosphatase inhibitors mixture. Proteins were separated by 10–12% SDS-PAGE and transferred to PVDF membranes. The membranes were blocked using 5% skim milk or BSA in 0.1% PBS-Tween for 1 hour at room temperature. The immunoblotting was performed using primary antibody against total *β*-catenin (# sc-7963, Santa Cruz Biotechnology, Santa Cruz, CA), active *β*-catenin clone 8E7 (# P35222, Millipore, Billerica, MA), total GSK-3*β* (# sc-9166, Santa Cruz Biotechnology, Santa Cruz, CA), p-Ser9-GSK-3*β* (# 93365, Cell Signaling, Danvers, MA), and the target genes cyclin D1, c-myc, and bcl-2 (# sc-717, sc-788, and sc-7382, respectively, Santa Cruz Biotechnology, Santa Cruz, CA) or fibronectin (# F3648, Sigma Aldrich, St. Louis, MO) and incubated overnight at 4°C. Proteins were detected using enhanced chemiluminescence techniques. The *β*-actin levels (# A1978, Sigma Aldrich, St. Louis, MO) were used as a load control and the densitometric analysis was performed using ImageJ (Wayne Rasband, National Institutes of Health, Bethesda, MD).

### 2.4. Immunostaining

Kidney tissue sections were fixed in Bouin's solution and embedded in Paraplast Plus. The immunohistochemistry and immunofluorescence were carried out in 5 *μ*m thick sections as previously done [21, 22]. Briefly, the tissue was dewaxed, rehydrated, rinsed in 0.05 mol/L Tris-phosphate-saline buffer pH 7.6, and then incubated overnight at 22°C with the primary antibody anti-collagen type III (Southern Biotech, Birmingham, AL), followed with appropriate secondary antibody and PAP complex (MP Biomedicals, Inc., Aurora, OH) was applied for 30 min. Samples incubated without primary antibody were used as negative control. Peroxidase activity was carried out with 0.1% (w/v) 3,3′-diaminobenzidine and 0.03% (v/v) hydrogen peroxide. Sections were counterstained with hematoxylin and then rehydrated, cleared with xylene, and mounted with Permount. Tissue sections were observed on a Nikon Eclipse E600 microscope and nonoverlapping images were photographed with a Nikon DS-Ri1 digital camera. For the immunofluorescence, the sections were incubated overnight at 4°C with the primary antibody anti-collagen type I or anti-OPN followed by Alexa Fluor 568 or 488, respectively (Invitrogen, Carlsbad, CA), and mounted with Vectashield (Vector Laboratories, Burlingame, CA). Nonoverlapping images were acquired using an Olympus BX51 fluorescence microscope and photographed with a Jenoptik ProgRes C5 digital camera. The stained area in each image was quantified utilizing computer-assisted image analysis software (Simple PCI, Hamamatsu). The values corresponding to total immunostained area were averaged and expressed as the mean absolute values per square micron and expressed as fold change compared to control values.

### 2.5. Statistical Analysis

The results are expressed as mean ± standard error (SEM). Differences between groups were assessed by a nonparametric Kruskal-Wallis test followed by Dunn's multiple range test. Statistical tests were performed using the GraphPad Prism software v 5.0 (GraphPad Software Inc., San Diego, CA). A probability of 95% (*P* < 0.05) was considered to be significant.

## 3. Results

### 3.1. Effect of ACE Inhibition on SBP and Renal Fibrosis in 2K1C Hypertensive Rats

Previously, the SBP through all experimental period was weekly evaluated in an independent group of 2K1C rats. SBP was significantly high in the third week after surgery (169 ± 4 mmHg, *P* < 0.05) compared to sham rats at the same time (121 ± 7 mmHg, *P* < 0.05). SBP of 2K1C rats continues to increase until fourth week (209 ± 4 mmHg) and remains high until eighth week (216 ± 8 mmHg) (Supplemental Figure S1 in Supplementary Material available online at http://dx.doi.org/10.1155/2015/726012).

**Figure 1:**
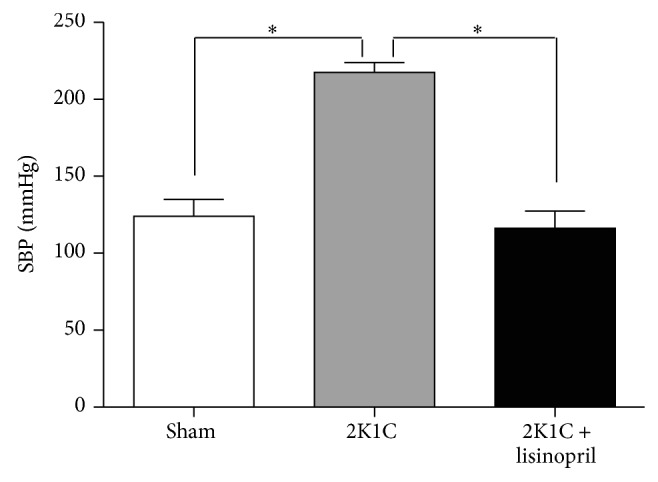
Effect of ACE inhibition on SBP in 2K1C rats. SBP in 2K1C treated with lisinopril was measured at the end of the experiment. The treatment with lisinopril decreased significantly the hypertension in 2K1C rats. The values represent mean ± SEM (*n* = 4 animals per group). ^∗^ *P* < 0.05 versus sham.

In our experimental conditions, the SBP significantly decreased in rats treated for 4 weeks with lisinopril compared with 2K1C rats without treatment (112 ± 5 versus 220 ± 4 mmHg, *P* < 0.05) (Figure 1).

**Figure 2:**
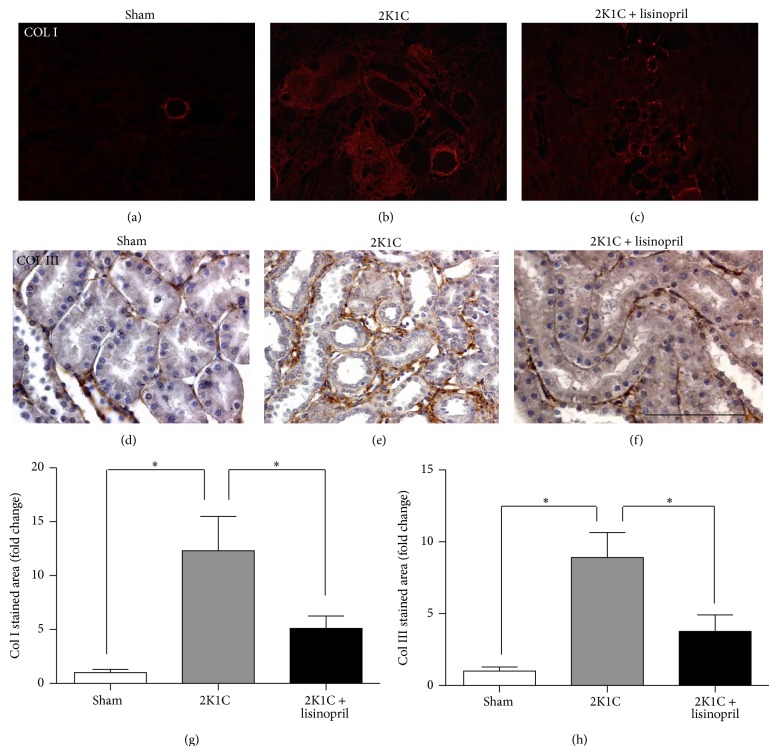
Effect of ACE inhibition on deposition of collagen types I and III in 2K1C hypertensive rats. Unclipped kidneys from 2K1C rats treated or not treated with lisinopril were immunostained for collagen types I and III. Representative immunofluorescence (IF) images for collagen type I of (a) sham, (b) 2K1C rats, or (c) 2K1C rats treated with lisinopril. Immunohistochemistry (IHQ) for collagen type III of (d) sham, (e) 2K1C rats, or (f) 2K1C rats treated with lisinopril. Quantification of (g) collagen type I and (h) collagen type III IHQ. Collagen types I and III immunostaining increases in 2K1C rats compared with the sham control, while the treatment with lisinopril decreases both. Scale bar = 100 *μ*m.

**Figure 3:**
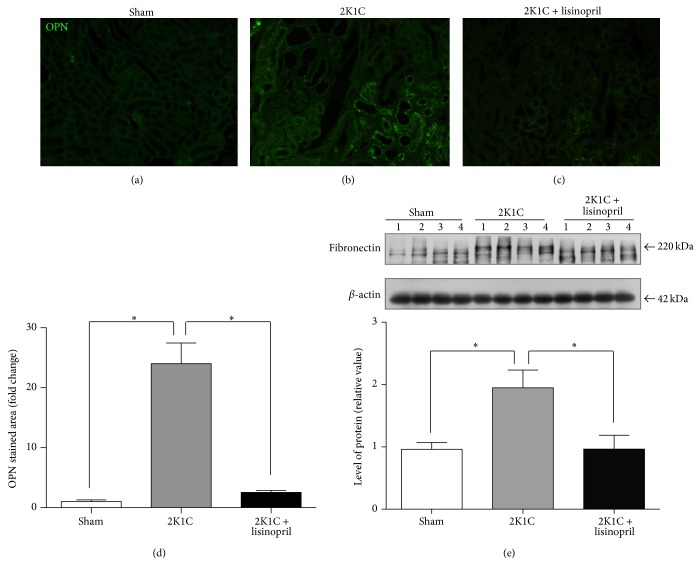
Effect of ACE inhibition on OPN and fibronectin levels in 2K1C hypertensive rats. Unclipped kidneys from 2K1C rats treated or not treated with lisinopril were immunostained for OPN and the protein level of fibronectin was evaluated by Western blot. (a) Sham, (b) 2K1C rats, or (c) 2K1C rats treated with lisinopril. (d) Quantification of OPN IF. (e) Representative Western blot and densitometric analysis of fibronectin. Numbers (1, 2, 3, and 4) in the Western blot indicate an individual animal sample in a given group. The OPN staining and fibronectin levels increase in 2K1C rats compared with the sham control whereas the treatment with lisinopril decreases both. Scale bar = 100 *μ*m. The values represent mean ± SEM (*n* = 4 animals per group). ^∗^ *P* < 0.05.

Renal fibrosis was evaluated by immunohistochemical staining for collagen type I, collagen type III, and OPN and the level of fibronectin protein by Western blot in the unclipped kidney. The unclipped kidneys from hypertensive rats showed a significant increased fibrosis assessed by deposition of collagen types I and III (Figures 2(a)-2(b), 2(d)-2(e), and 2(g)-2(h)) and it was associated with a significant increase in OPN immunostaining (Figures 3(a)–3(d)) and fibronectin protein level (Figure 3(e)). The treatment of 2K1C hypertensive rats for four weeks with lisinopril significantly reduced the immunostaining for collagen types I and III (Figures 2(c), 2(f), and 2(g)) and OPN (Figures 3(c) and 3(d)) together with a decrease in the fibronectin protein level (Figure 3(e)).

### 3.2. Lisinopril Treatment Inhibits the *β*-Catenin Signaling Pathway in 2K1C Hypertensive Rats

**Figure 4:**
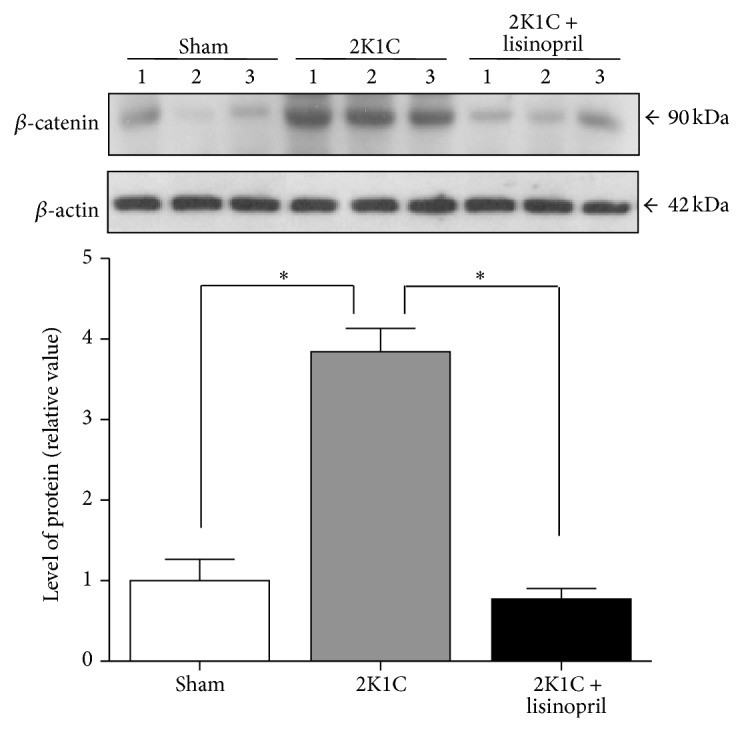
Protein levels of *β*-catenin in 2K1C hypertensive rats treated with lisinopril. Western blot analysis of *β*-catenin in the unclipped kidney from 2K1C rats treated or not treated with lisinopril was performed. The hypertension induced an increase in the levels of *β*-catenin, while the treatment with lisinopril decreased it. Numbers (1, 2, and 3) in the Western blot indicate an individual animal sample in a given group. The level of protein was normalized to *β*-actin levels and was expressed as a fold change relative to sham rats. The bars represent the mean ± SEM (*n* = 4). ^∗^ *P* < 0.05.

**Figure 5:**
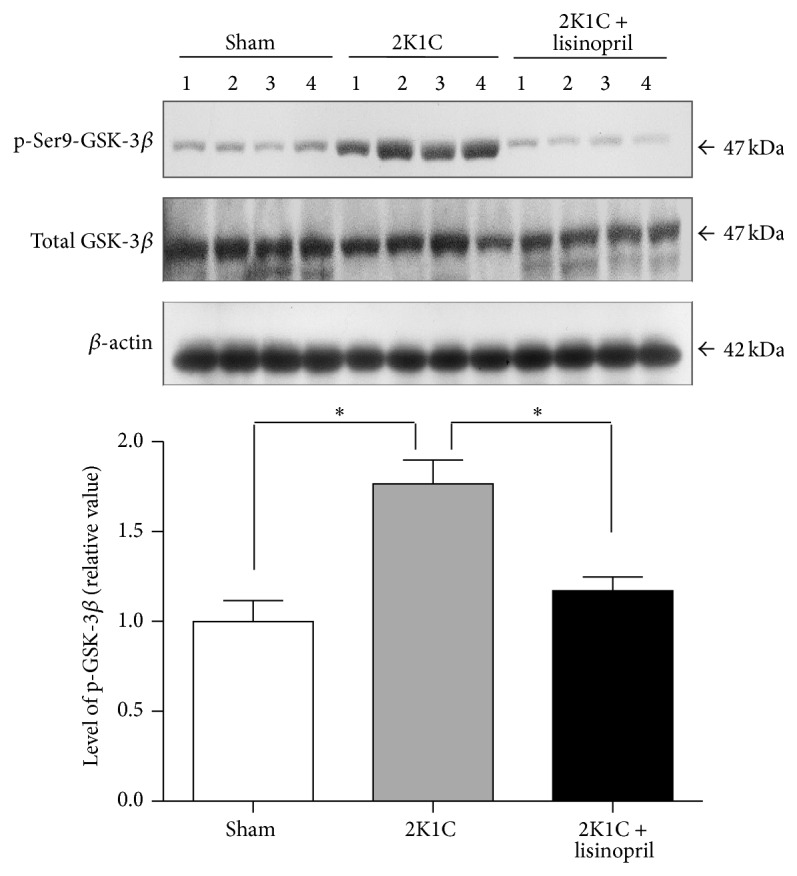
Levels of p-Ser9-GSK-3*β* in 2K1C hypertensive rats treated with lisinopril. Western blot analysis of total GSK-3*β* and p-Ser9-GSK-3*β* in the unclipped kidney from 2K1C rats treated or not treated with lisinopril was performed. The unclipped kidney showed an induction in the phosphorylation of Ser9-GSK-3*β*; the treatment with lisinopril reversed this effect on the GSK-3*β* phosphorylation. The level of protein was normalized to total GSK-3*β* and *β*-actin levels and the ratio was expressed as relative units normalized to sham rats. Numbers (1, 2, 3, and 4) in the Western blot indicate an individual animal sample in a given group. The bars represent the mean ± SEM (*n* = 4). ^∗^ *P* < 0.05.

**Figure 6:**
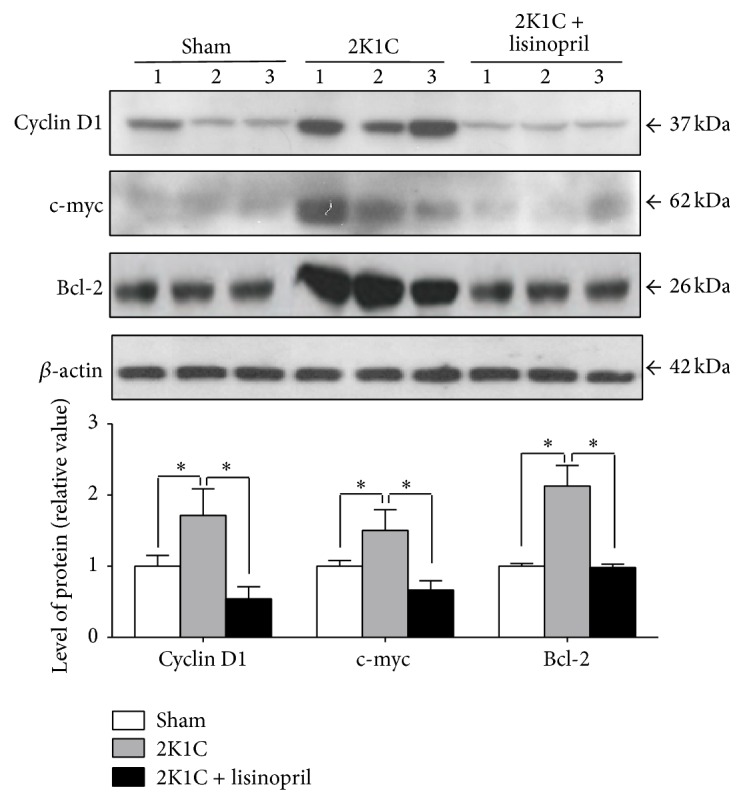
Protein levels of *β*-catenin-dependent gene products in 2K1C rats treated with lisinopril. Western blot analysis of cyclin D1, c-myc, and bcl-2 in the unclipped kidney from 2K1C rats treated or not treated with lisinopril was done. The protein levels of target of Wnt signaling cyclin D1, c-myc, and bcl-2 were increased in unclipped kidney. Lisinopril reverses this effect on the protein levels in all of them. The level of protein was normalized to *β*-actin levels and the ratio was expressed as relative units normalized to sham rats. Numbers (1, 2, and 3) in the Western blot indicate an individual animal sample in a given group. The bars represent the mean ± SEM (*n* = 4). ^∗^ *P* < 0.05.

To assess the status of the *β*-catenin signaling in 2K1C hypertensive rats and the effect of ACE inhibition, we evaluated the protein level of several components of the *β*-catenin signaling pathway by Western blot analysis in the unclipped kidney from 2K1C Goldblatt rats without treatment or treated with lisinopril. The protein levels of the *β*-catenin were significantly increased in unclipped kidney from hypertensive rats when compared to sham (*P* < 0.05, Figure 4). Interestingly, treatment with lisinopril restored the level of *β*-catenin to control values (Figure 4). Furthermore, we studied the expression of inactive form (p-Ser9) of GSK-3*β* and our results showed an increase in p-Ser9-GSK-3*β* in the unclipped kidney from 2K1C hypertensive rats compared to sham (Figure 5). The treatment with lisinopril significantly reduced this effect on GSK-3*β* phosphorylation (Figure 5, *P* < 0.05). Moreover, we evaluated the levels of the classic *β*-catenin-dependent target genes such as cyclin D1, c-myc, and bcl-2. Our results indicated an increase in the level of cyclin D1, c-myc, and bcl-2 in the unclipped kidney from 2K1C hypertensive rats compared to sham (Figure 6). Interestingly, inhibition of ACE significantly reduced the protein levels of all of them (Figure 6).

### 3.3. Inhibition of *β*-Catenin Signaling Reduced Renal Fibrosis in 2K1C Hypertensive Rats

**Figure 7:**
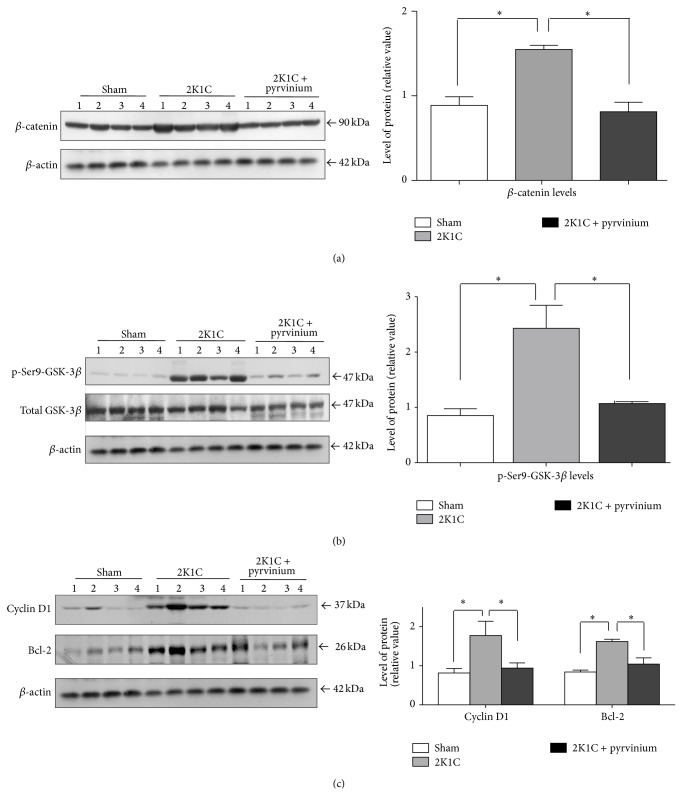
Inactivation of *β*-catenin signaling pathway in 2K1C rats by treatment with pyrvinium pamoate. The analysis of the status of different components of the Wnt signaling pathways by Western blot in the unclipped kidney from 2K1C Goldblatt rats treated with pyrvinium pamoate was performed. The levels of (a) *β*-catenin, (b) p-Ser9-GSK-3*β*, and (c) the target genes cyclin D1 and bcl-2 decrease after the treatment with pyrvinium pamoate in total extracts from unclipped kidney, indicating that the signaling is inactive. The level of protein was normalized to *β*-actin levels and the ratio was expressed as relative units normalized to sham rats. Numbers (1, 2, 3, and 4) in the Western blot indicate an individual animal sample in a given group. The bars represent the mean ± SEM (*n* = 4). ^∗^ *P* < 0.05.

**Figure 8:**
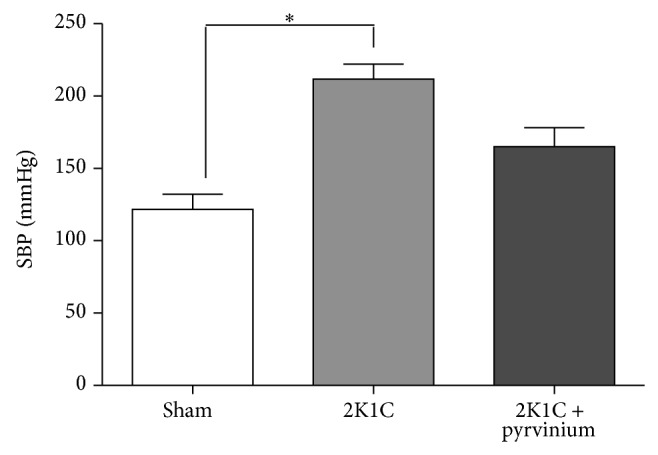
Effect of pyrvinium pamoate on SBP in 2K1C hypertensive rats. The SBP in 2K1C hypertensive rats treated with pyrvinium pamoate was measured. The treatment with pyrvinium pamoate did not reduce significantly the levels of SBP observed in 2K1C rats. The values were expressed as mean ± SEM (*n* = 4). ^∗^ *P* < 0.05.

**Figure 9:**
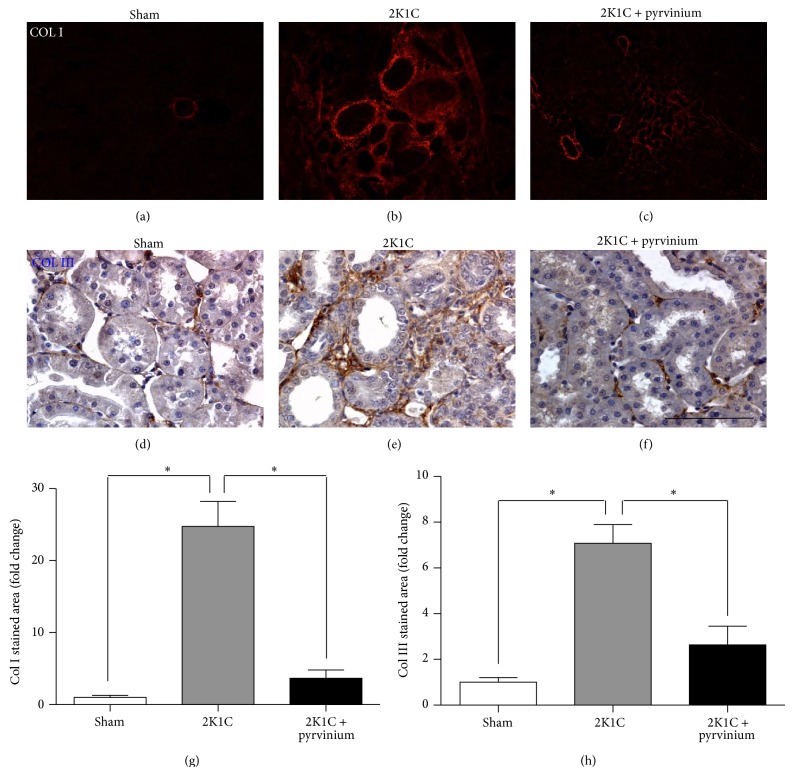
Effect of pyrvinium pamoate on deposition of collagen types I and III in 2K1C hypertensive rats. Unclipped kidneys from 2K1C rats treated with pyrvinium pamoate were immunostained for collagen type I and type III. Representative images from immunofluorescence for collagen type I of (a) sham, (b) 2K1C rats, and (c) 2K1C rats treated with pyrvinium pamoate. Immunohistochemistry for collagen type III of (d) sham, (e) 2K1C rats, and (f) 2K1C rats treated with lisinopril. Quantification of (g) collagen type I IF and (h) collagen type III IHQ. The treatment with pyrvinium pamoate decreased both collagen type I and type III in the unclipped kidney from 2K1C rats compared. Scale bar = 100 *μ*m.

**Figure 10:**
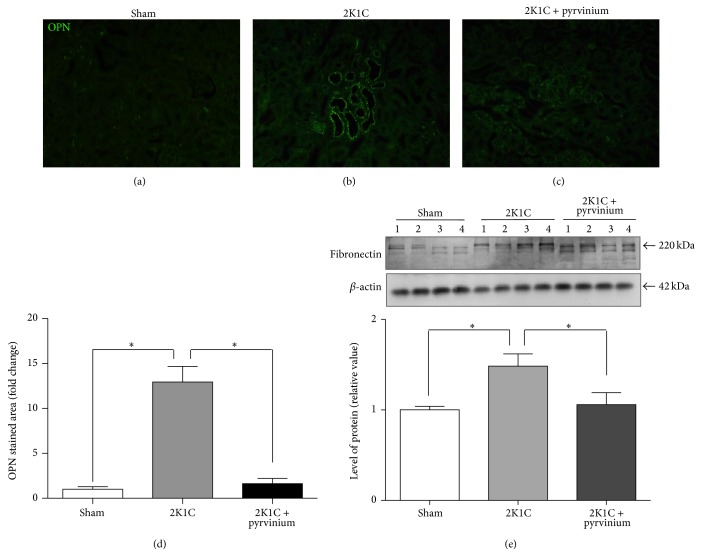
Effect of pyrvinium pamoate on the levels of OPN and fibronectin in 2K1C hypertensive rats. Unclipped kidneys from 2K1C rats treated with pyrvinium pamoate were immunostained for OPN and the levels of fibronectin were evaluated by Western blot. (a) Sham, (b) 2K1C rats, and (c) 2K1C rats treated with pyrvinium pamoate. (d) Quantification of OPN IF. (e) Western blot of fibronectin protein levels normalized to *β*-actin levels. Numbers (1, 2, 3, and 4) in the Western blot indicate an individual animal sample in a given group. The sections from unclipped kidney from 2K1C hypertensive rats treated with pyrvinium pamoate show a reduction of both immunofluorescence of OPN and fibronectin protein level in total kidney extracts. Scale bar = 100 *μ*m. The values represent the mean ± SEM (*n* = 4). ^∗^ *P* < 0.05.

In order to test the hypothesis whether the *β*-catenin signaling is playing a role in the fibrosis we inhibited this signaling pathway using pyrvinium pamoate. The results showed that pyrvinium pamoate treatment significantly reduced the level of total *β*-catenin protein (Figure 7(a)) which is consistent with the reduction of the phosphorylation of GSK-3*β* in the inhibitory residue serine 9 (Figure 7(b)). Furthermore, in accordance with these results we observed a reduction of the expression of *β*-catenin-dependent gene products, cyclin D1 and bcl-2 (Figure 7(c)). We observed that the treatment with pyrvinium pamoate reduced SBP; nevertheless, this decrease was not statistically significant (170 ± 3 versus 212 ± 6, *P* > 0.05, Figure 8). Immunostaining for collagen types I and III in renal tissue from unclipped kidney shows that inhibition of the *β*-catenin signaling significantly reduced the level of collagen types I and III (Figures 9(b)-9(c), 9(e)-9(f) and 9(g)-9(h)); similar reduction was observed in OPN levels as visualized by immunofluorescence (Figures 10(a)–10(d)) and also observed in fibronectin level (Figure 10(e)) compared to 2K1C hypertensive rats.

## 4. Discussion

Our study demonstrated that *β*-catenin signaling pathway is activated and could play an important role in the development of kidney fibrosis secondary to hypertension in 2K1C hypertensive rats. We showed that treatment with an ACEi reduced the *β*-catenin signaling pathway along with a reduction of SBP and renal fibrosis compared with untreated hypertensive rats. Furthermore, we demonstrated that treatment with pyrvinium pamoate inhibits *β*-catenin signaling pathway and reduced renal fibrosis in hypertensive rats.

The 2K1C model is an established model of hypertension and it is dependent on the increased activity of RAS [5]. In accordance with previous studies, we recorded a continuous increase in SBP reaching a plateau after four weeks of surgery [23, 24]. Hypertension-induced renal failure is a progressive event associated with kidney remodeling characterized by fibrosis and alterations of renal function [25]. Our results indicated that 2K1C-induced hypertension was associated with renal fibrosis induction assessed by the increase in the levels of collagen types I and III, OPN, and fibronectin. Importantly, our data indicate that the increased collagen types I and III, fibronectin, and OPN levels in kidney from hypertensive rats were reduced after ACE inhibition, indicating that the RAS signaling is an important factor in the development of hypertension and fibrosis and also supporting the early notion from Guan et al. [26] that high levels of Ang II in the unclipped kidney result from enhanced ACE activity. The antifibrotic properties of ACEi have been shown in other models of renal fibrosis [27] as it is known that overactivation of RAS, mainly by the action of Ang II, is an important contributor to the pathogenesis of hypertension and it has profibrotic effects that contribute to the progression of chronic kidney disease [7].

Several pathways have been described as contributors in this process, including TGF-*β*/Smad signaling [28] as a main effector of Ang II-induced injury. However, the molecular mechanism remains unknown and the researches for effective treatments are still under development. Thus, search for new signal pathways and development of new therapeutic strategies are in progress. Recent studies have demonstrated that aberrant *β*-catenin signaling plays a key role in the development of organ fibrosis, suggesting it may be a novel therapeutic target in fibrotic disorders [29]. In agreement with previous studies showing the activation of *β*-catenin signaling in other models of renal injury [16], our data showed the increase of specific components of the *β*-catenin signaling in 2K1C hypertensive rats. Our results showed an increase in *β*-catenin levels together with the inhibition of GSK-3*β* and an increase in *β*-catenin-dependent gene products in the nonclipped kidney, suggesting the activation of this pathway in hypertensive rats. Interestingly, the upregulation of most of these gene products has been previously showed in obstructive uropathy renal damage model (UUO) [30, 31] and they participate in processes such as regulation of the cell cycle, cell proliferation, and apoptosis, among others. In addition, it has been showed that fibronectin [17], collagen types I and III [19, 32], and OPN [18] are all *β*-catenin target genes; therefore the upregulation of all these genes might account for the important role of *β*-catenin-dependent signaling in the control of adult tissue renewal and fibrosis. Despite that, we do not show regulation of the transcription factor TCF/LEF in our model; the upregulation of LEF-1 and TCF transcription factor has been demonstrated in the UUO rats [33, 34] and the colocalization of *β*-catenin with the transcription factor LEF-1 in the nuclei of podocytes has been shown in rats with focal glomerulosclerosis [35]; therefore it is conceivable that in 2K1C rats the interaction of *β*-catenin with TCF/LEF transcription factors could mediate the upregulation of its target genes.

On the other hand, as we mentioned previously, there is wide evidence suggesting that TGF-*β* is one of the main effectors of the Ang II-induced renal damage [10]. Using 2K1C model, Chen et al. (2011) [30] showed a decrease in the high levels of TGF-*β* in the nonclipped kidney after the treatment with enalapril. Coincidentally, the cross talk and cooperation between the Wnt/*β*-catenin signaling and TGF-*β* signaling pathways in the fibrotic processes have been showed [13, 25, 36]. Therefore, it is conceivable that the *β*-catenin signaling is a common effector of different pathways activated by Ang II to promote fibrosis in the kidney.

It is worth noting that our data showed that the ACE inhibition in 2K1C hypertensive rats was associated with an inhibition of the *β*-catenin signaling, suggesting a modulator role for RAS in this pathway. The levels of *β*-catenin, p-Ser9-GSK-3*β*, and the *β*-catenin-dependent gene products decreased after the treatment with lisinopril, suggesting that Ang II can stimulate the *β*-catenin signaling to promote the fibrosis in 2K1C hypertensive rats. A cross talk between the Ang II signaling and the *β*-catenin pathway appears to occur, since Ang II modulates GSK-3*β* phosphorylation inducing fibrosis in the heart [37]. However, additional experiments are needed to elucidate how Ang II modulates the *β*-catenin signaling pathway in the kidney. Recently, Zhou et al. (2015) [38] reported that all RAS genes are novel target genes of Wnt/*β*-catenin signaling pathway activation; it could suggest that the activation of this pathway contributes to maintaining the overactivation of RAS in chronic kidney disease.

Our results are in agreement with previous studies in UUO model of renal damage showing that inhibition of the Wnt/*β*-catenin signaling reduced renal *β*-catenin accumulation and decreased fibrosis [16, 39]. In our study, the inhibition of *β*-catenin signaling pathway reduces renal fibrosis in the unclipped kidney from 2K1C hypertensive rats. Furthermore, a novel finding in our study was the effect of pyrvinium pamoate (an FDA-approved drug) on reducing renal fibrosis in the unclipped kidneys. Recently, it was shown that pyrvinium pamoate may have therapeutic benefit in two different models of myocardial remodeling [40, 41] and here we are showing evidence of its potential therapeutic use in the hypertensive renal disease. Although Thorne et al. [20] showed that the *β*-catenin signaling was potently targeted by pyrvinium pamoate through the activation of casein kinase 1, novel mechanisms of action of pyrvinium pamoate have been suggested in different cancer cells lines, identifying pyrvinium pamoate as a novel anticancer drug able to target mitochondrial respiration in hypoglycemic/hypoxic conditions [42] and to inhibit the unfolded protein response induced by glucose starvation [43] and a noncompetitive androgen receptor inhibitor in prostate cancer cell lines. Despite the fact that our data strongly suggest that pyrvinium pamoate inhibits the *β*-catenin-dependent signaling pathway in 2K1C rats, the possibility that pyrvinium pamoate could also affect other cellular processes cannot be ruled out and future studies using a different *β*-catenin inhibitor may answer these questions.

Interestingly, our data showed that the treatment of hypertensive rats with pyrvinium pamoate treatment tends to decrease SBP; however, this decrease was not statistically significant; therefore, the animals remain exposed to high blood pressure levels. It is known that Ang II contributes to fibrotic lesions by the direct activation of profibrotic pathways in the kidney [7]. Furthermore, in UUO, which is a normotensive model of renal damage, Satoh et al. [44] showed that the Wnt/*β*-catenin signaling pathway is active in tubular cells as early as day 3 after UUO, and the activation of Wnt/*β*-catenin signaling pathway occurs independently of the increased blood pressure. Additional studies are required to better understand the contribution of the *β*-catenin signaling in the fibrotic lesions of unclipped kidney, in particular a Wnt/*β*-catenin signaling inhibitor without effect on blood pressure. However, we have recently provided evidence that* in vitro* Ang II induced the expression of profibrotic factors through the *β*-catenin-dependent signaling in mouse collecting duct cells. Ang II upregulated the *β*-catenin protein levels together with GSK-3*β* phosphorylation and *β*-catenin target genes. Interestingly, all these effects were prevented by pyrvinium pamoate, indicating that, in M-1 collecting duct cells, the *β*-catenin signaling pathway mediates the stimulation of fibrotic factors in response to AT1 receptor activation [36] independently of changes in blood pressure.

In summary, our findings suggest that *β*-catenin signaling pathway is active in fibrotic process in the unclipped kidney from 2K1C hypertensive rats. The inhibition of *β*-catenin signaling pathway by pyrvinium pamoate and lisinopril decreases renal fibrosis in 2K1C hypertensive rats. These findings provide a better understanding of the mechanisms involved in renal damage in hypertension and open new therapeutic approaches to control the fibrosis induced by hypertension.

## Supplementary Material

With the purpose of monitoring the level of systolic blood pressure (SBP) throughout the all experimental period (0-8 weeks) we performed an independent group of 2K1C and sham rats (n=8 rats per group) and the SBP was measured at 0, 3, 4 and 8 weeks after surgery. A significant increase on SBP was observed from the 3rd week after surgery. (p<0.05)
