# Supplementary material for: β-Catenin-Dependent Signaling Pathway Contributes to Renal Fibrosis in Hypertensive Rats
Source: Biomed Res Int. 2015 Apr 7;2015:726012. doi: 10.1155/2015/726012 (PMC4405227; doi:10.1155/2015/726012)
Supplement: Supplementary file 1 — With the purpose of monitoring the level of systolic blood pressure (SBP) throughout the all experimental period (0-8 weeks) we performed an independent group of 2K1C and sham rats (n=8 rats per group) and the SBP was measured at 0, 3, 4 and 8 weeks after surgery. A significant increase on SBP was observed from the 3rd week after surgery. (p<0.05) [file 726012.f1.pdf]

726012 v2 accepted for publication

Supplementary Figure

β-catenin-dependent signaling pathway contributes to renal fibrosis in hypertensive rats

Catherina A. Cuevas[1], Cheril Tapia-Rojas[2], Carlos Cespedes[1], Nibaldo C. Inestrosa[2,3] and Carlos P. Vio[1,3].

[1]Department of Physiology, [2]Department of Cellular and Molecular Biology, Faculty of Biological Sciences, [3]Center for Aging and Regeneration CARE Chile UC, Pontificia Universidad Catolica de Chile, Alameda 340, Santiago, Chile.

Corresponding author :
Carlos P. Vio, M.D.
Department of Physiology
Pontificia Universidad Catolica de Chile
Alameda 340, Santiago, Chile
e-mail: cvio@uc.cl

With the purpose of monitoring the level of systolic blood pressure (SBP) throughout the all experimental period (0-8 weeks) we performed an independent group of 2K1C and sham rats (n= 8 rats per group) and the SBP was measured at 0, 3, 4 and 8 weeks after surgery. A significant increase on SBP was observed from the 3rd week after surgery. ($p<0.05$)

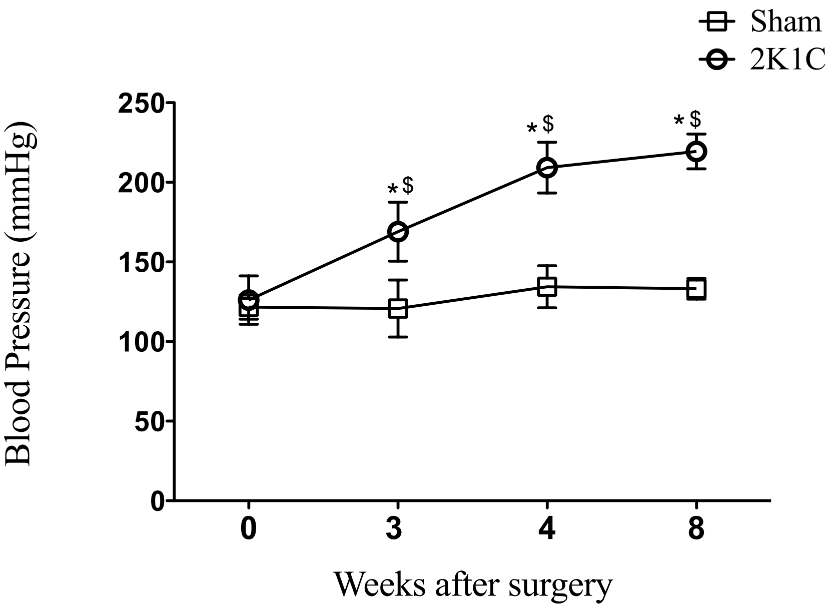

Supplementary Fig. 1 Time course of SBP in 2K1C rats. The SBP was measured in sham rats (control) and 2K1C hypertensive rats at 0, 3, 4 and 8 weeks after surgery. A significant increase on SBP was recorded in 3rd week after surgery.*$P<0.05$
